# EEGGAN-Net: enhancing EEG signal classification through data augmentation

**DOI:** 10.3389/fnhum.2024.1430086

**Published:** 2024-06-21

**Authors:** Jiuxiang Song, Qiang Zhai, Chuang Wang, Jizhong Liu

**Affiliations:** ^1^School of Advanced Manufacturing, Nanchang University, Nanchang, Jiangxi, China; ^2^Shaoxing Institute of Advanced Research, Wuhan University of Technology, Shaoxing, Zhejiang, China; ^3^Xiangyang Auto Vocational Technical College, Intelligent Manufacturing College, Xiangyang, Hubei, China

**Keywords:** brain-computer interface, electroencephalography, Conditional Generative Adversarial Network, cropped training, Squeeze-and-Excitation attention

## Abstract

**Background:**

Emerging brain-computer interface (BCI) technology holds promising potential to enhance the quality of life for individuals with disabilities. Nevertheless, the constrained accuracy of electroencephalography (EEG) signal classification poses numerous hurdles in real-world applications.

**Methods:**

In response to this predicament, we introduce a novel EEG signal classification model termed EEGGAN-Net, leveraging a data augmentation framework. By incorporating Conditional Generative Adversarial Network (CGAN) data augmentation, a cropped training strategy and a Squeeze-and-Excitation (SE) attention mechanism, EEGGAN-Net adeptly assimilates crucial features from the data, consequently enhancing classification efficacy across diverse BCI tasks.

**Results:**

The EEGGAN-Net model exhibits notable performance metrics on the BCI Competition IV-2a and IV-2b datasets. Specifically, it achieves a classification accuracy of 81.3% with a kappa value of 0.751 on the IV-2a dataset, and a classification accuracy of 90.3% with a kappa value of 0.79 on the IV-2b dataset. Remarkably, these results surpass those of four other CNN-based decoding models.

**Conclusions:**

In conclusion, the amalgamation of data augmentation and attention mechanisms proves instrumental in acquiring generalized features from EEG signals, ultimately elevating the overall proficiency of EEG signal classification.

## 1 Introduction

Stroke stands as a predominant contributor to enduring disabilities in the contemporary world (Feigin et al., 2019; Khan et al., 2020). The reinstatement of motor function is imperative for stroke survivors to execute their daily tasks. Nonetheless, stroke inflicts damage on the central nervous system, impacting all aspects of motor control. Consequently, certain patients find themselves unable to independently perform motor recovery activities, with some even incapable of executing upper limb movements due to the severity of the condition. In response to this challenge, a substantial number of stroke patients rely on physiotherapists who manually guide their arm movements to facilitate physical recovery (Claflin et al., 2015). However, this intervention approach not only proves inefficient but also entails considerable labor costs. Against this backdrop, some scholars advocate for the integration of the motor imagery paradigm to aid in the recovery of stroke patients.

The motor imagery paradigm, involving mental simulation and replication of movement, holds promise in enhancing muscle memory, fortifying neural pathways, and ultimately refining motor performance (Leamy et al., 2014; Benzy et al., 2020). Recent years have experienced a surge in interest in the motor imagery paradigm, primarily due to its distinctive feature of involving mental simulation without actual physical actions (Zhang et al., 2016; Roy, 2022). Within the medical realm, researchers have harnessed this paradigm to decode patients’ electroencephalogram (EEG) signals for diverse applications, including wheelchair control (Huang et al., 2019), prosthetic limb manipulation (Kwakkel et al., 2008), and exoskeleton operation (Gupta et al., 2020) to cursor control (Abiri et al., 2020), spelling, and converting thoughts to text (Makin et al., 2020). Expanding beyond the medical sphere, motor imagery tasks have found utility in non-medical domains, spanning vehicle control (Jafarifarmand and Badamchizadeh, 2019; Hekmatmanesh et al., 2022), drone manipulation (Chao et al., 2020), smart home applications (Zhong et al., 2020; Zhuang et al., 2020), security systems (Landau et al., 2020), gaming (Lalor et al., 2005; Liao et al., 2012), and virtual reality endeavors (Lohse et al., 2014). Despite the versatility of motor imagery tasks across these domains, their adoption encounters limitations owing to limited classification accuracy.

The use of EEG for motor imagery tasks encounters challenges due to its low signal-to-noise ratio. Consequently, the extraction of key features from EEG signals becomes a crucial aspect of motor imagery classification. Researchers have explored various research avenues, spanning from traditional machine learning feature extraction methods to more contemporary deep learning approaches.

Common methods for feature extraction from traditional EEG signals include common spatial pattern (CSP) (Yang et al., 2016), filter bank common spatial pattern (FBCSP) (Zhang et al., 2021), principal component analysis (PCA) (Kundu and Ari, 2018), and independent component analysis (ICA) (Karimi et al., 2017). Al-Qazzaz et al. (2021) proposed a multimodal feature extraction method, the AICA-WT-TEF algorithm, which extracts time-domain, entropy-domain, and frequency-domain features, then fuses them to enhance the model’s classification accuracy. Similarly, Al-Qazzaz et al. (2023) introduced a feature enhancement method involving the calculation of fractal dimension (FD) and Hurst exponent (HUr) as complexity features, and Tsallis entropy (TsEn) and dispersion entropy (DispEn) as irregularity parameter features, thereby improving the model’s classification accuracy.

In recent years, the convolutional neural network (CNN) has been thrust into the limelight, largely propelled by the advent of deep learning (Lashgari et al., 2021; Fu et al., 2023; Liu K. et al., 2023). A notable contribution in EEG signal classification within this field is EEGNet (Lawhern et al., 2018), a concise CNN that enhances spatial features among individual EEG channels through intricate convolution and separable convolution structures. This design has yielded exceptional outcomes in various EEG signal classification tasks. To address the acknowledged limitations of EEGNet’s weak global information extraction capability, EEGATCNet, a structure derived from EEGNet, featuring multiple attention heads, was introduced by Liu M. et al. (2023) to compensate for this deficiency. Recognizing the temporal nature of EEG signals, Peng et al. (2022) proposed TIE-EEGNet, an EEGNet structure augmented with temporal features. By integrating a structure for temporal feature extraction into EEGNet, a synergistic amalgamation of spatial and temporal features is achieved, thereby enhancing the overall model accuracy.

In the realm of deep learning models, the amount of available data plays a pivotal role in determining performance. However, EEG tasks are hindered by a scarcity of data due to the high cost of acquisition, acquisition difficulties, and privacy concerns associated with data sharing. To mitigate this challenge, Fahimi et al. (2021) leveraged conditional Deep Convolutional Generative Adversarial Networks (DCGANs) to generate raw EEG signal data, resulting in improved classification model accuracy. Likewise, Zhang et al. (2020) employed DCGANs to generate EEG time-frequency maps via short-time Fourier transform, and Xu et al. (2022) used DCGANs for generating EEG topographic maps via Modified S-Transform, both contributing to enhanced model accuracy. Notably, Raoof and Gupta (2023) not only devised a conditional GAN structure for 1-dimensional EEG generation but also incorporated KL divergence and KS test metrics to validate the reliability of the generated data from the final model.

While GANs facilitate data generation and expand dataset sizes, they introduce noise features during training data generation. To address this challenge and enhance the accuracy of EEG signal classification tasks, we propose a novel approach that combines GAN data enhancement, a cropped training strategy, and the Squeeze-and-Excitation (SE) attention mechanism. This combination forms the basis of our data enhancement-based EEG signal classification model, named EEGGAN-Net. Hence, the primary contributions of our study are as follows:

A. We introduce a robust data enhancement strategy that utilizes Conditional Generative Adversarial Network (CGAN) enhanced data, implements a cropped training strategy, and integrates the SE attention mechanism to discern crucial features, thereby enhancing EEG signal classification.
B. The proposed model is systematically benchmarked against contemporary EEG signal classification models, demonstrating superior performance.
C. The efficacy of each component within EEGGAN-Net is rigorously confirmed through ablation experiments.

The subsequent sections unfold as follows: Section “2 EEGGAN-Net” provides an in-depth elucidation of the complete EEGGAN-Net framework. Section “3 Experiments” comprehensively details the experiments, results, and discussions. Finally, Section “4 Conclusion and future work” furnishes conclusive remarks and outlines potential avenues for future research.

## 2 EEGGAN-Net

### 2.1 Model structure

**FIGURE 1:**
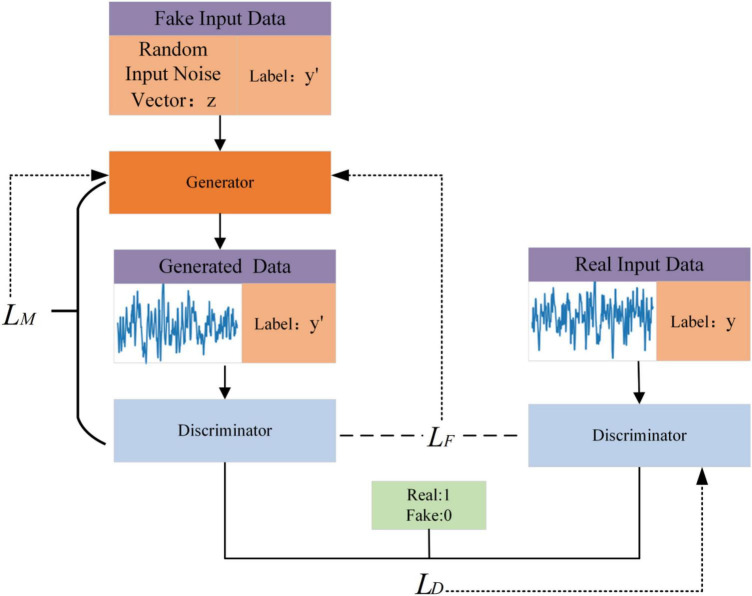
The structure of EEGGAN-Net.

EEGGAN-Net consists of three main parts: generator, discriminator and classifier, and the overall structure is shown in Figure 1. Initially, the generator processes noise input to produce a set of synthetic data. These synthetic data, alongside authentic samples, are then fed into the discriminator for training, enabling it to distinguish between real and synthetic samples. This iterative process between the generator and discriminator continues until a stable generator model is achieved, ensuring consistent generation of synthetic samples. Concurrently, the classifier, essentially the trained discriminator, is utilized to classify the augmented EEG signal training set.

The loss function of EEGGAN-Net comprises two primary components: the generator loss and the discriminator loss. Within the generator loss, there are two constituents: *L_M_* and *L_F_*, while the discriminator loss is denoted as *L_D_*. These two components of the loss function are backpropagated separately. The dashed line with arrows indicates the association with their respective losses, where *L_M_* represents the basic generator loss; *L_F_* is the feature matching loss, and *L_D_* denotes the discriminator loss.

#### 2.1.1 Generator

**FIGURE 2:**
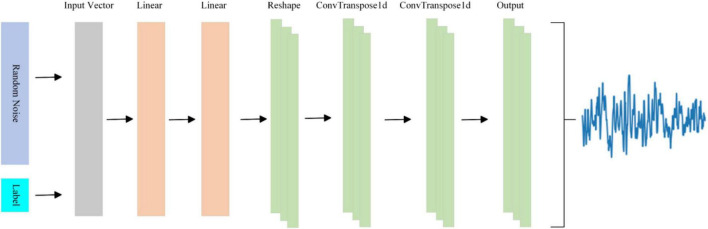
The structure of generator.

In the architecture of EEGGAN-Net, the primary function of the generator is to process random noise and labels, mapping them onto a data space resembling authentic data associated with corresponding labels. The generator’s structure is schematically shown in Figure 2.

Within the generator, the input vector is composed of a fusion of random noise and labels. These input vectors traverse through a sequential arrangement of two fully connected layers, one Reshape layer, two ConvTranspose1d layers, and ultimately an output layer, resulting in the generation of EEG signals. Specifically, the fully-connected layer is instrumental in generating pivotal feature points, the ConvTranspose1d layer is employed for the creation of multi-channel EEG signals, the Reshape layer facilitates the connection between the fully-connected and ConvTranspose1d layers. And the output layer is similar to the ConvTranspose1d layer, but differs slightly in the following ways: the activation function is different, and the output layer does not have an intermediate normalization layer. The specifics of the generator’s architecture are summarized in Table 1.

**TABLE 1:** Detailed architecture of the generator.

Layers	Hidden dimension	Output dimension	Activation
Input		(101)	
Linear	256	(256)	
BatchNorm		(256)	LeakyReLU
Linear	936	(936)	
BatchNorm		(936)	LeakyReLU
Reshape		(1, 936)	
ConvTranspose1d	64 (64)	(64, 999)	
BatchNorm		(64, 999)	LeakyReLU
ConvTranspose1d	64 (32)	(32, 1062)	
BatchNorm		(32, 1062)	LeakyReLU
Output	64 (C)	(C, 1125)	Tanh

#### 2.1.2 Discriminator

**FIGURE 3:**
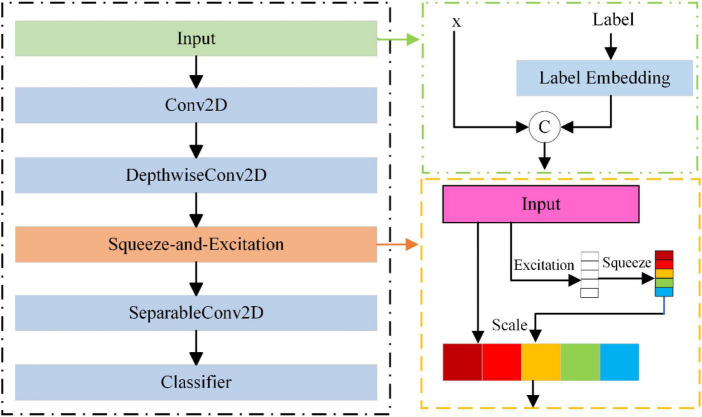
The structure of discriminator.

The primary function of the discriminator is to distinguish whether the input data originates from an authentic data distribution or if it is synthetic data produced by the generator. To enhance the discriminator’s precision and incentivize the generator to generate more lifelike data, this study introduces the EEGCNet network structure. EEGCNet initiates its architecture by considering the interrelations among various channels of EEG signals, corresponding to the different leads of the EEG cap. It incorporates the SE attention module onto the foundation of EEGNet. The schematic structure of the discriminator is shown in Figure 3.

EEGCNet comprises distinct modules: Input, Conv2d, DepthwiseConv2D, SE, SeparableConv2D, and Classifier. The Input module consists of two components: the input data, denoted as x, and the label that has undergone encoding. The Conv2d module serves as a temporal filter, extracting frequency information of various magnitudes by configuring the convolution kernel size. The DepthwiseConv2D module functions as a spatial filter, extracting spatial features between individual channels of the EEG signals. The SE attention module explicitly models the interdependence among channels, adaptively recalibrating the channels by explicitly representing the interdependencies between them. The SeparableConv2D module serves as a feature combiner, optimizing the amalgamation of features from each kernel in the time dimension. Finally, the Classifier module is responsible for classifying these features. The details of the discriminator are summarized in Table 2.

**TABLE 2:** Detailed architecture of the discriminator.

Layers	Filter	Size	Output dimension	Activation	Options
Input			(1, C, T+1)		
Conv2D	F_1_	(1, 64)	(F_1_, C, T+1)		Mode = same
BatchNorm			(F_1_, C, T+1)		
DepthwiseConv2D	D*F_1_	(C, 1)	(D*F_1_, 1, T+1)		Mode = valid, depth = D
BatchNorm			(D*F_1_, 1, T+1)	ELU	
AvgPool2d		(1, 4)	(D*F_1_, 1, (T+1)//4)		
Dropout			(D*F_1_, 1, (T+1)//4)		*p* = 0.25
SE			(D*F_1_, 1, (T+1)//4)		
SeparableConv2D	F_2_	(1, 16)	(F_2_, 1, (T+1)//4)		Mode = same
BatchNorm			(F_2_, 1, (T+1)//4)	ELU	
AvgPool2d		(1, 8)	(F_2_, 1, (T+1)//32)		
Dropout			(F_2_, 1, (T+1)//32)		*p* = 0.25
Flatten			(F_2_*((T+1)//32))		
Classifier		1	(1)		

*Indicates the multiplication of two values.

#### 2.1.3 Classifier

The primary function of the classifier is to ascertain the motor imagery category, relying on the input EEG signal. In the architecture of EEGGAN-Net, a pivotal modification is made by substituting the last layer of the discriminator’s structure with a motor imagery classification layer, thereby facilitating the integration of the classifier. Subsequently, both raw training data and generated data undergo ingestion into the classifier for the purpose of model training and the subsequent derivation of the classifier model.

### 2.2 Cropped training

**FIGURE 4:**
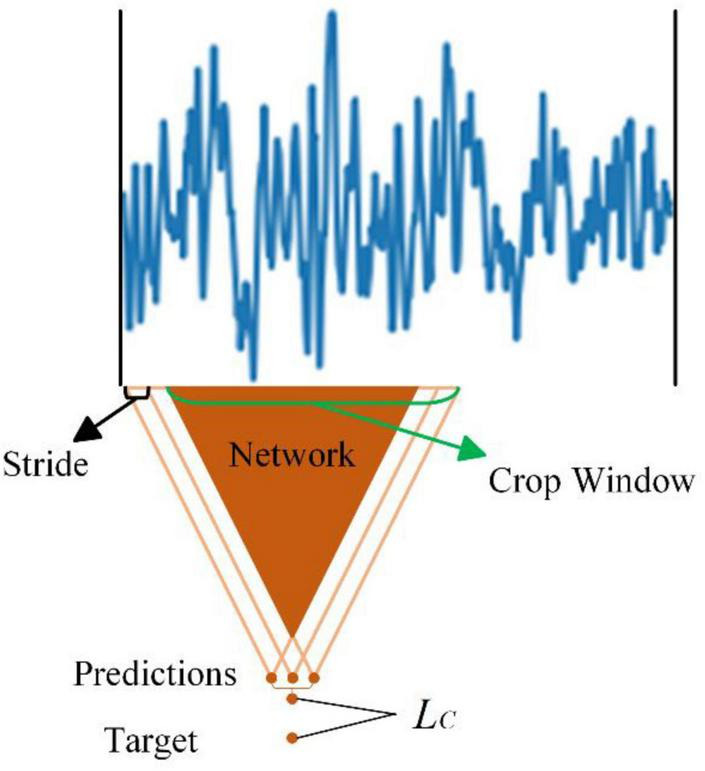
The operation of the cropped training.

To enhance the classification performance of our model, we employ a training strategy known as cropped training for the classifier. Originally introduced by Schirrmeister et al. (2017) in the context of EEG decoding, cropped training extends the model training dataset through a sliding window, thereby leveraging the entire spectrum of features present in EEG signals. This approach is particularly suitable for convolutional neural networks (CNNs) as their receptive fields tend to be localized. The process of cropped training primarily takes place within the discriminator, wherein data is selectively trimmed to enhance the model’s proficiency in identifying overarching features. And the operation of the cropped training is shown in Figure 4.

The size of the cropped window dictates the dimensions of the sliding window, while the step size determines the number of sliding windows. For instance, considering the initial dataset with 22 channels and 1,125 time-point data, setting the sliding window size to 500 time sample points and the step size to 125 time sample points results in the expansion of the data from (1, 22, 1125) to six (1, 22, 500) data samples. Importantly, these newly generated data samples share identical labels. Thus, the implementation of cropped training significantly augments the available dataset, effectively magnifying the size of the training set by a factor of 6, despite the high similarity among these additional data samples.

### 2.3 Loss function

The model’s loss function comprises three primary components: the generator loss function, discriminator loss function, and classifier loss function.

#### 2.3.1 Generator loss function

The generator loss function encompasses two segments: the fundamental generator loss function and the feature matching loss function. Its calculation formula is shown in Equation 1.


(1) $$L_{G}=L_{M}+\lambda *L_{F}$$

Where, *L_G_* denotes the generator loss; *L_M_* represents the basic generator loss; *L_F_* is the feature matching loss, and λ signifies the weight of the feature matching loss term (default is 0.5).

Basic generator loss function: This measures the mean squared error between the data generated by the generator and the target output. It guarantees the generator produces realistic data, deceiving the discriminator in the process. The calculation formulas for the basic generator loss function are presented in Equations 2–4.


(2) $$L_{M}=\frac{1}{n}\,\sum_{i=1}^{n}{\left(D\,\left(G\,\left(z_{i},l_{g_{i}}\right),l_{g_{i}}\right)-v_{i}\right)}^{2}$$

(3) $$z_{i}=F\,\left(\mu ,\sigma ,n_{d}\right)$$

(4) $$l_{g_{i}}=r\,a\,n\,d\,\left(n_{c}\right)$$

Where, *n* is the number of samples; *z_i_* and *l*_*g_i_*_ denote randomly generated data and corresponding labels for the *i*-th sample; μ and σ are the mean and variance of the original data; *F* represents the function generating random arrays based on random numbers and variance; *n_d_* is the dimensions of the generated arrays; *rand*(*n*_*c*_) denote rand generates a random integer in the range of *n_c_*, where *n_c_* is the number of categories in the original data; *G* is the generator; *D* is the discriminator; and *v_i_* is the target output corresponding to the *i*-th sample, representing the true label (denoted as 1 in this paper).

Feature matching loss function: This assesses the feature similarity between the generated image and the real image. By minimizing the feature matching loss, the generator is incentivized to generate images similar to the real data in terms of intermediate layer features, thereby enhancing the quality and diversity of the generated images. The Feature Matching Loss Function is calculated as shown in Equation 5.


(5) $$L_{F}=\frac{1}{n}\,\sum_{i=1}^{n}\sum_{j=1}^{m}{\left(R_{i\,j}-G_{i\,j}\right)}^{2}$$

Where, *m* is the number of features; *R*_*ij*_ denotes the *j*-th feature of the *i*-th real data, and *G*_*ij*_ denotes the *j*-th feature of the *i*-th generated data.

#### 2.3.2 Discriminator loss function

The discriminator loss function calculates the classification error on real data and generated data, facilitating the discriminator in distinguishing between generated and real data. The discriminant loss function is calculated as shown in Equation 6.


(6) $$L_{D}=\frac{\sum_{i=1}^{n}{\left(D\,\left(x_{i},l_{i}\right)-v_{i}\right)}^{2}+\sum_{i=1}^{n}{\left(D\,\left(G\,\left(z_{i},l_{g_{i}}\right),l_{g_{i}}\right)-f_{i}\right)}^{2}}{2\,n}$$

Where, *L_D_* denotes the discriminator loss; *x_i_* and *l_i_* are the *i*-th real data and corresponding category, respectively; *f_i_* represents the target output corresponding to the *i*-th sample, a tensor representing the generated labels (denoted as 0 in this paper).

#### 2.3.3 Classifier loss function

Aligned with the training strategy, the classifier loss function is CroppedLoss, which computes the loss function between predicted categories and actual categories. Predicted categories are determined by the average classification probability of the cropped data samples. CroppedLoss is calculated as shown in Equation 7.


(7) $$L_{C}=F_{L}\,\left(a\,v\,g\,\_\,p\,r\,e\,d\,s,l\,a\,b\,e\,l\right)$$

(8) $$a\,v\,g\,\_\,p\,r\,e\,d\,s=\frac{\sum_{i=1}^{n}p\,r\,e\,d\,s}{n}$$

Where, *avg*_*preds* denotes the labels corresponding to the average prediction probability, calculated by Equation 8. *n* indicates that an original EEG signal is cropped to obtain *n* cropped EEG signals. In Equation 8, *preds* denotes the result obtained for each cropped sample input to the classifier.

## 3 Experiments

The EEG signals underwent preprocessing through bandpass filtering and normalization, followed by feature extraction and classification. Specifically, a 200-order Blackman window bandpass filter was applied to the raw EEG data, with the bandpass filtering interval set at (Benzy et al., 2020; Altaheri et al., 2023) Hz as outlined in this paper.

The experimental procedures were conducted within the PyTorch framework, utilizing a workstation equipped with an Intel(R) Xeon(R) Gold 5117 CPU @ 2.00 GHz and an Nvidia Tesla V100 GPU.

### 3.1 Dataset

The BCI Competition IV 2a dataset constitutes a motor imagery dataset with four distinct categories: left hand, right hand, foot, and tongue movements. This dataset was derived from nine subjects across two sessions conducted on different dates, designated as training and test sets. Each session task comprised 288 trials, with 72 trials allocated to each movement category. In every trial, subjects were presented with an arrow pointing in one of four directions (left, right, down, or up), corresponding to the intended movement (left hand, right hand, foot, or tongue). The cue, displayed as the arrow, persisted for 4 s, during which subjects were instructed to mentally visualize the associated movement. The overall duration of each trial averaged around 8 s.

The BCI Competition IV 2b dataset represents a motor imagery dataset with two distinct categories, corresponding to left- and right-handed movements. This dataset comprises data collected across a total of five sessions, involving nine subjects. Notably, the initial three sessions encompass training data, while the subsequent two sessions consist of test data. It is crucial to highlight that the dataset exclusively features EEG data from three specific channels: C3, Cz, and C4. For more comprehensive details regarding this dataset, kindly refer to the following URL: https://www.bbci.de/competition/iv/desc_2b.pdf.

Data extraction for subsequent processing focused on a specific temporal window, precisely from 0.5 s after the cue onset to its conclusion, resulting in a duration of 4.5 s. It is noteworthy that the preceding two datasets have been partitioned into a training set and a test set internally, hence they will not be reiterated later on. And the BCI Competition IV 2a dataset employs 22 channels, while the BCI Competition IV 2b dataset utilizes 3 channels. This discrepancy delineates the format of the input data for the EEGGAN-Net as (22,1125) or (3,1125).

### 3.2 Similarity evaluation

The assessment of similarity was employed to quantify the impact of GAN enhancement on model performance. This evaluation utilized the KS test and KL scatter to gauge the likeness between real EEG signals and their enhanced counterparts.

The KS test serves as a metric for comparing the cumulative distribution function (CDF) of real and enhanced data. In our analysis, the output is derived by subtracting 1 from the D statistic of the KS test, signifying the maximum distance between authentic and generated EEG signals. It is noteworthy that we employed the Inverse Kolmogorov–Smirnov Test. Consequently, lower output values indicate a higher similarity between real EEG data and their enhanced counterparts.

KL divergence, on the other hand, is employed to quantify the disparity between the distribution of real data and that of the enhanced data. Hence, a diminished KL divergence value suggests a greater similarity between the two distributions. Table 3 presents the outcomes of the similarity assessment for the EEGGAN-Net generated data.

**TABLE 3:** The outcomes of the similarity assessment for the EEGGAN-Net generated data.

Dataset	KS test	KL divergence
BCI Competition IV 2a	0.8516	0.7415
BCI Competition IV 2b	0.9327	0.8114

### 3.3 Model comparison

**FIGURE 5:**
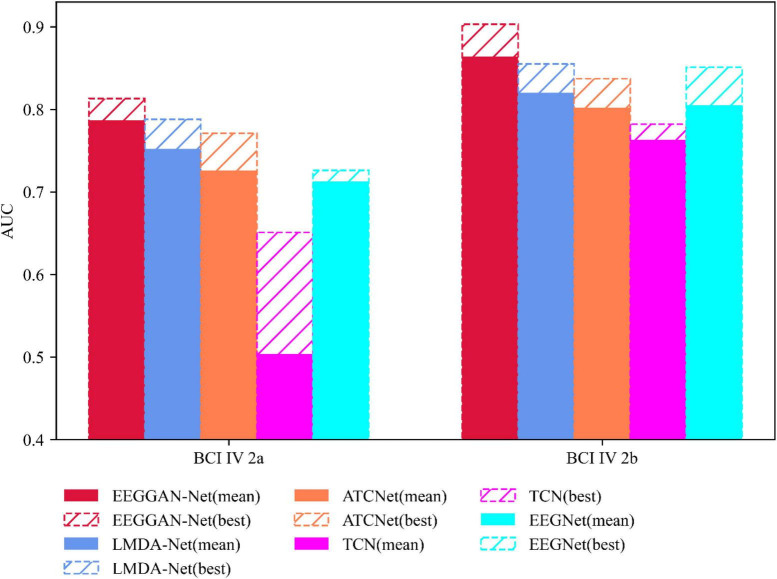
Classification performance of competitive models under ACC metrics.

**FIGURE 6:**
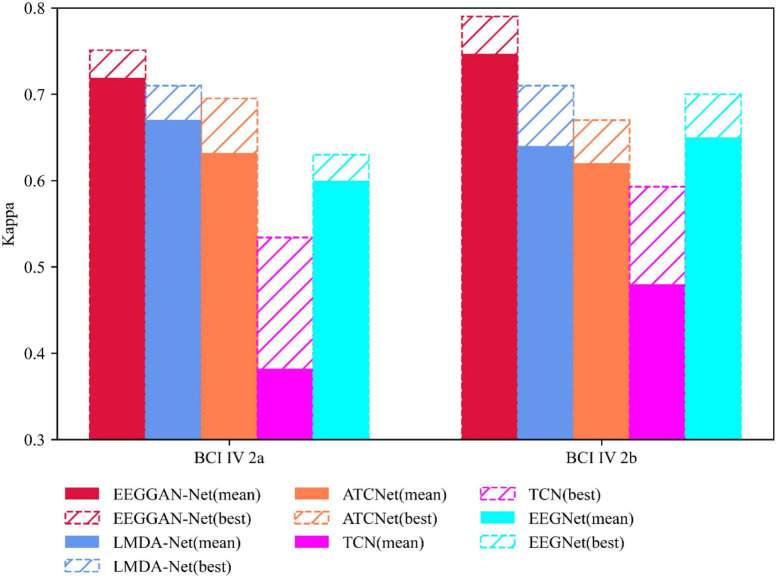
Classification performance of competing models under Kappa metrics.

Under identical testing conditions, we conducted twenty repetitions of evaluations to assess the performance of EEGGAN-Net, LMDA-Net (Miao et al., 2023), ATCNet (Altaheri et al., 2023), TCN (Ingolfsson et al., 2020), and EEGNet. Figures 5, 6 show the accuracy (ACC) and Kappa metrics for the above models using the BCI IV 2a dataset and the BCI IV 2b dataset, respectively. In these figures, “best” indicates the performance with the most favorable results across repeated tests, while “mean” represents the average performance over multiple repetitions.

Figure 5 illustrates that EEGGAN-Net exhibits the highest classification accuracy across both datasets, achieving 81.3 and 90.3%. Additionally, the prediction volatility is remarkably low, maintaining an average accuracy of 78.7 and 86.4%. This underscores the superior classification performance of EEGGAN-Net compared to other models.

Conversely, in the same context, EEGNet demonstrates significantly lower effectiveness when compared to EEGGAN-Net, with average accuracies of 71.3 and 80.5%. Furthermore, its maximum accuracies reach only 72.6 and 85.1% in the respective datasets.

Similarly, Figure 6 demonstrates that EEGGAN-Net has the highest Kappa values of 0.751 and 0.79, while predicting the least volatility, with average Kappa of 0.719 and 0.747, respectively.

These results collectively highlight EEGGAN-Net’s superior accuracy and stability compared to other models. The notably lower volatility positions EEGGAN-Net as a highly effective candidate for deployment in online brain-computer interface (BCI) applications.

### 3.4 Wilcoxon signed-rank tests

To verify whether there are significant differences between EEGGAN-Net and each of the other methods, we perform the Wilcoxon signed-rank tests on the performance values of tested models. The results of significance tests are listed in Table 4. “+*” and “++” signify that EEGGAN-Net is statistically better than the compared algorithm under consideration at a significant level of 0.1 and 0.05, respectively. “+” denotes that EEGGAN-Net is only quantitatively better. One can see that EEGGAN-Net can achieve statistically superior performance in most of the cases, which demonstrates EEGGAN-Net is highly effective as compared to the other algorithms.

**TABLE 4:** *p*-values of Wilcoxon signed-rank tests for the average performance comparisons between EEGGAN-Net and other models.

	BCI IV 2a		BCI IV 2b	
	ACC	Kappa	ACC	Kappa
LMDA-Net	1.2500E-03++	4.3750E-03++	8.7017E-04++	3.7514E-02++
ATCNet	8.1250E-02+*	3.1042E-04++	0.6217+	5.4225E-02+*
TCN	6.2500E-04++	4.4212E-05++	4.4233E-02++	2.5427E-03++
EEGNet	1.4413E-03++	1.1342E-05++	7.1561E-04++	1.6459E-02++

+ and * is used as a symbol to indicate a range of significance levels, as described in the text.

### 3.5 Ablation experiments

**FIGURE 7:**
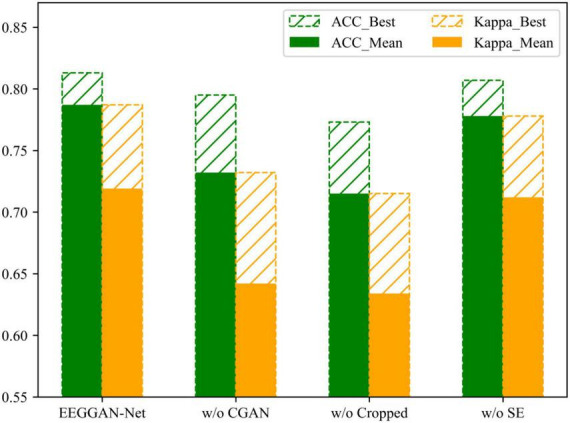
The results of ablation experiments under the BCI IV 2a dataset.

**FIGURE 8:**
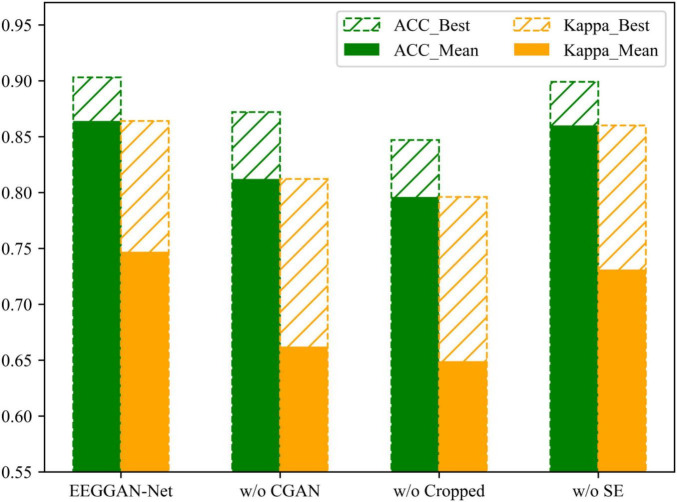
The results of ablation experiments under the BCI IV 2b dataset.

We performed an ablation analysis to further investigate the effectiveness of the CGAN data enhancement, cropped training and SE attention in EEGGAN-Net. We sequentially studied the deleted CGAN data enhancement, cropped training and SE attention and compared them with EEGGAN-Net. The experimental results are shown in Figures 7, 8. In the figure, w/o means without.

#### 3.5.1 CGAN data enhancement

CGAN data enhancement is a key step in EEGGAN-Net. The original training dataset is expanded through data enhancement, which in turn improves the classification accuracy of the model. To prevent model overfitting, the generated data is set to be half of the original data. Also, to prevent class imbalance from affecting the model, the number of each class generated is set to be the same.

Figures 7, 8 demonstrate the comparative results of the CGAN data augmentation ablation experiment. When CGAN data augmentation was removed, the performance of EEGGAN-Net decreased, which illustrates that CGAN data augmentation can effectively increase the number of available training samples. Meanwhile, the stability of EEGGAN-Net also decreased after removing the CGAN data enhancement. It further illustrates the importance of data samples for deep learning models, and data augmentation is a better way to improve the model classification accuracy and stability.

#### 3.5.2 Cropped training

Although CGAN data enhancement can expand the original data samples, the expansion is often accompanied by some noise features, while the cropped training strategy makes the model focus more on the key features in each EEG segment by sliding averaging over the time window.

The comparative results of the ablation experiments with cropped training are shown in Figures 7, 8. As mentioned above, when trimming training is removed, the performance of EEGGAN-Net is worse than removing CGAN data augmentation, and the reason behind this is that CGAN data augmentation expands the dataset while introducing noise features. It also illustrates that cropped training allows the model to focus on the global features of the data, which improves the classification accuracy of the model.

#### 3.5.3 SE attention

The comparative results of the ablation experiments of the SE attention module are shown in Figures 7, 8. Although the SE attention module can extract the interrelationships between individual channels and thus assign different weights, the experimental results show that the removal of the SE attention module slightly decreases the effectiveness of EEGGAN-Net. This further indicates that: assigning different weights to each channel has a certain effect on classification, but there exists a certain correlation between the individual EEG channels, and it is this correlation that causes the effect of assigning weights to be less effective than expected.

### 3.6 Discussion

Combining the conclusions in Sections “3.3 Model comparison,” “3.4 Wilcoxon signed-rank tests,” and “3.5 Ablation experiments,” EEGGAN-Net enhances the accuracy and stability of EEG signal classification by integrating CGAN data augmentation, cropped training, and SE considerations, outperforming existing models.

The pivotal role of data is underscored in deep learning, where both quantity and quality significantly impact classification accuracy. This principle extends to EEGGAN-Net, where a multitude of data enhancement strategies, such as CGAN data generation and sliding time window expansion, align with deep learning model strategies. Experimental results affirm the efficacy of data augmentation, albeit acknowledging its nuanced challenges. For instance, CGAN data generation introduces noise features, demanding robust feature extraction capabilities, and expanding data through sliding time windows risks overfitting, leading to a cautious approach in EEGGAN-Net, limiting data expansion to six times the original dataset, a conservative measure in the context of EEG data.

Similarly, the attention mechanism proves effective. While the attention mechanism globally extracts features and emphasizes important data elements, its application in EEG data confronts challenges stemming from the inherent complexity and correlation of temporal features, compounded by noise from various sources. The SE attention mechanism in EEGGAN-Net deviates from conventional approaches by enhancing model classification through differential weighting between channels, thereby mitigating the impact of noise.

Despite the commendable results of the EEGGAN-Net model, it shares common vulnerabilities with GANs, particularly susceptibility to parameter fluctuations during training, leading to challenges in generating realistic data. Notably, EEGGAN-Net distinguishes itself by leveraging the stability and high accuracy inherent in the EEGNet-based discriminator structure, obviating the need for frequent adjustments, which stands as an improvement over traditional GANs in EEG signal classification.

## 4 Conclusion and future work

In this study, we present the EEGGAN-Net architecture as an innovative approach to improve the accuracy of classifying EEG signals during motor imagery tasks. Our devised training strategy utilizes CGAN data augmentation, complemented by cropped training to extract overarching features from the data. Additionally, we incorporate the SE attention module to discern relationships among individual EEG channels. Through a synergistic integration of data augmentation and attention mechanisms, our model is equipped to identify generalized features within EEG signals, thereby enhancing overall classification efficacy. Comparative evaluations against five prominent classification models demonstrate that EEGGAN-Net achieves the highest classification accuracy and stability. Furthermore, through ablation experiments, we elaborate on and validate the impact of each architectural component on the experimental outcomes.

In future research, we will focus on further optimizing the CGAN training strategy to improve model efficiency. Concurrently, we plan to explore the application of the model in facilitating patient recovery in real-world brain-computer interface scenarios.

## Data availability statement

The original contributions presented in this study are included in this article/supplementary material, further inquiries can be directed to the corresponding author.

## Ethics statement

Ethical review and approval was not required for the study on human participants in accordance with the local legislation and institutional requirements. Written informed consent from the patients/participants or patients’/participants’ legal guardian/next of kin was not required to participate in this study in accordance with the national legislation and the institutional requirements.

## Author contributions

JS: Formal analysis, Methodology, Software, Writing – original draft, Writing – review & editing. QZ: Supervision, Writing – review & editing. CW: Resources, Writing – review & editing. JL: Resources, Writing – review & editing.
